# Killing of Pseudomonas aeruginosa by Chicken Cathelicidin-2 Is Immunogenically Silent, Preventing Lung Inflammation *In Vivo*

**DOI:** 10.1128/IAI.00546-17

**Published:** 2017-11-17

**Authors:** Maarten Coorens, Brandon J. H. Banaschewski, Brandon J. Baer, Cory Yamashita, Albert van Dijk, Henk P. Haagsman, Ruud A. W. Veldhuizen, Edwin J. A. Veldhuizen

**Affiliations:** a Department of Infectious Diseases and Immunology, Division of Molecular Host Defence, Faculty of Veterinary Medicine, Utrecht University, Utrecht, The Netherlands; b Department of Physiology and Pharmacology, the University of Western Ontario, London, Ontario, Canada; University of California San Diego School of Medicine

**Keywords:** innate immunity, cathelicidin, host defense peptide, immunomodulation, alternative to antibiotics

## Abstract

The development of antibiotic resistance by Pseudomonas aeruginosa is a major concern in the treatment of bacterial pneumonia. In the search for novel anti-infective therapies, the chicken-derived peptide cathelicidin-2 (CATH-2) has emerged as a potential candidate, with strong broad-spectrum antimicrobial activity and the ability to limit inflammation by inhibiting Toll-like receptor 2 (TLR2) and TLR4 activation. However, as it is unknown how CATH-2 affects inflammation *in vivo*, we investigated how CATH-2-mediated killing of P. aeruginosa affects lung inflammation in a murine model. First, murine macrophages were used to determine whether CATH-2-mediated killing of P. aeruginosa reduced proinflammatory cytokine production *in vitro*. Next, a murine lung model was used to analyze how CATH-2-mediated killing of P. aeruginosa affects neutrophil and macrophage recruitment as well as cytokine/chemokine production in the lung. Our results show that CATH-2 kills P. aeruginosa in an immunogenically silent manner both *in vitro* and *in vivo*. Treatment with CATH-2-killed P. aeruginosa showed reduced neutrophil recruitment to the lung as well as inhibition of cytokine and chemokine production, compared to treatment with heat- or gentamicin-killed bacteria. Together, these results show the potential for CATH-2 as a dual-activity antibiotic in bacterial pneumonia, which can both kill P. aeruginosa and prevent excessive inflammation.

## INTRODUCTION

Pseudomonas aeruginosa is a Gram-negative bacterium which can cause opportunistic infections in the lungs of susceptible patients (1–3). Chronic P. aeruginosa infections are commonly associated with cystic fibrosis (CF) and chronic obstructive pulmonary disease (COPD), and effective treatment is difficult due to the development of multidrug resistance (MDR) in these bacteria (4–6). Adding to the complexity of the pathophysiology of the infected CF and COPD patients is the presence of chronic inflammation within the lung (7, 8). This chronic inflammation is characterized by high neutrophil numbers and the release of proinflammatory mediators, which are insufficient to clear the infection. The tissue damage and lung dysfunction associated with chronic infection are ultimately the most common cause of death in these patients (9–11).

Research into novel therapeutics for the treatment of P. aeruginosa infections has shown that cathelicidins are a promising alternative to conventional antibiotics (12–15). Cathelicidins are short cationic peptides with broad-spectrum antimicrobial activity against various pathogens, including Gram-positive and Gram-negative bacteria (16, 17). This broad-spectrum antimicrobial activity has also been observed for chicken cathelicidin-2 (CATH-2) and includes activity against MDR P. aeruginosa strains (15). In addition, unlike most other cathelicidins, CATH-2 has been shown to retain antimicrobial activity under physiological conditions (18). Importantly, we recently showed that CATH-2 has a dual function, with regard to both killing Gram-negative bacteria and subsequently inhibiting the inflammatory response against the killed microbe (19). This “silent killing” was demonstrated against Escherichia coli and Salmonella enterica serovar Enteritidis, where CATH-2 neutralizes lipopolysaccharide (LPS) and lipoproteins released from the bacterial outer membrane, which prevents Toll-like receptor 2 (TLR2) and TLR4 activation on macrophages. However, it is unknown whether CATH-2 is able to silently kill other clinically relevant Gram-negatives, such as P. aeruginosa, and whether this reduced inflammation is also observed in an *in vivo* situation.

This study tests the hypothesis that CATH-2 mediates silent killing of P. aeruginosa both *in vitro* and *in vivo*. To test silent killing *in vitro*, tumor necrosis factor alpha (TNF-α) and interleukin 6 (IL-6) release by murine macrophages was determined after stimulation with CATH-2-killed P. aeruginosa and was compared to release after stimulation with viable, heat-killed, and gentamicin-killed P. aeruginosa. Subsequently, the *in vivo* effect of CATH-2-killed P. aeruginosa on leukocyte recruitment and release of cytokines in the bronchoalveolar lavage fluid (BALF) was determined after intratracheal instillation in mice. Overall, this study demonstrates CATH-2-mediated silent killing of P. aeruginosa in both *in vitro* and *in vivo* settings and underlines the potential therapeutic value of CATH-2-based anti-infectives.

## RESULTS

### CATH-2 inhibits P. aeruginosa-induced macrophage activation.

**FIG 1:**
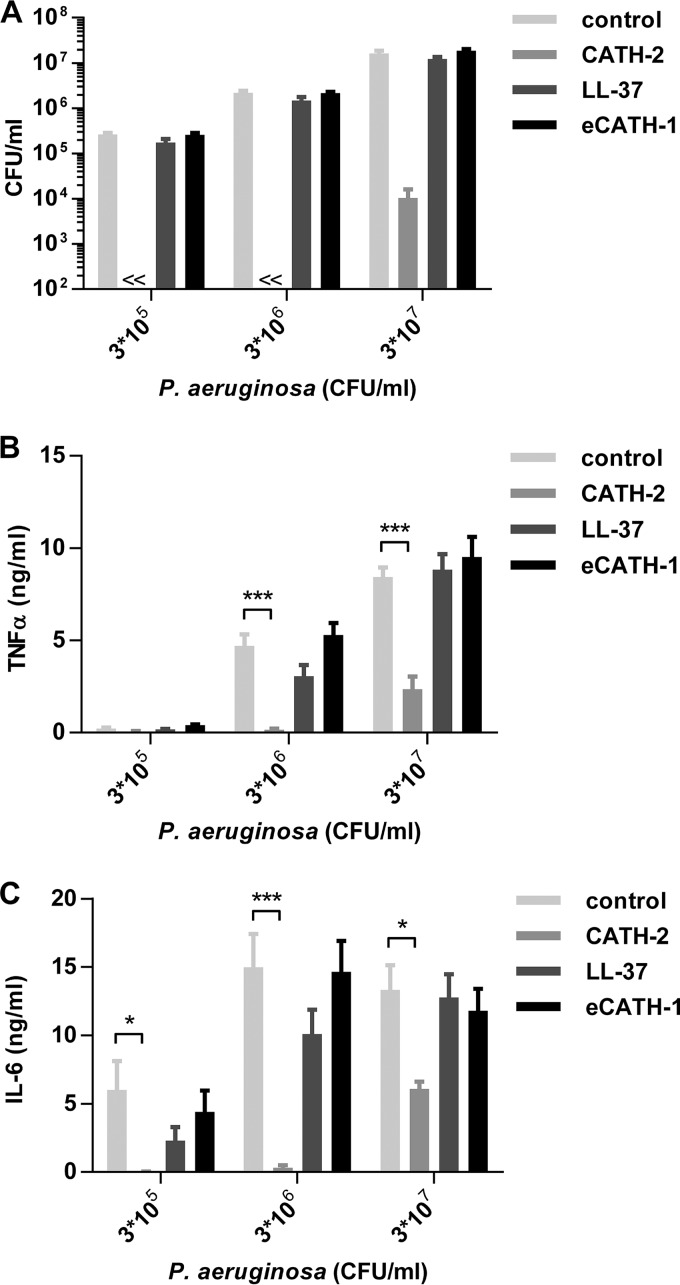
CATH-2 inhibits P. aeruginosa-induced macrophage activation. (A) Various concentrations of P. aeruginosa were incubated with 5 μM CATH-2, LL-37, or eCATH-1 in DMEM plus 10% FCS for 2 h at 37°C, after which the viability was assessed by colony counting assays. (B and C) J774.A1 cells were stimulated with various concentrations of P. aeruginosa in combination with 5 μM CATH-2, LL-37, or eCATH-1 in DMEM plus 10% FCS for 2 h at 37°C, followed by a double wash and incubation for an additional 22 h in DMEM plus 10% FCS plus 250 μg/ml gentamicin. TNF-α production (B) was determined after 2 h, while IL-6 production (C) was determined after 24 h. *n* = 3 or more. Error bars = standard errors of the mean (SEM). Statistical differences are determined by two-way analysis of variance (ANOVA) with Bonferroni's *post hoc* test. *, *P* < 0.05; **, *P* < 0.01; ***, *P* < 0.001.

To determine the antimicrobial activity of CATH-2 against P. aeruginosa under physiological cell culture conditions, a colony counting assay was performed in Dulbecco modified Eagle medium (DMEM) plus 10% fetal calf serum (FCS) (Fig. 1A). The activity of CATH-2 was compared to that of the human antimicrobial peptide LL-37 and equine CATH-1 (eCATH-1). A 5 μM concentration of CATH-2 completely killed 3 × 10^5^ to 3 × 10^6^ CFU/ml P. aeruginosa and decreased P. aeruginosa viability 1,000-fold at 3 × 10^7^ CFU/ml. In contrast, LL-37 and eCATH-1 did not show any antimicrobial activity. To determine whether CATH-2-mediated killing resulted in reduced macrophage activation by P. aeruginosa, J774.A1 murine macrophages were stimulated with viable P. aeruginosa in combination with 5 μM CATH-2, LL-37, or eCATH-1, after which TNF-α production (Fig. 1B) and IL-6 production (Fig. 1C) were determined after 2 h and 24 h, respectively. CATH-2 significantly reduced P. aeruginosa-induced TNF-α and IL-6 production, in contrast to LL-37 and eCATH-1, which did not affect cytokine production.

### CATH-2 silently kills P. aeruginosa.

**FIG 2:**
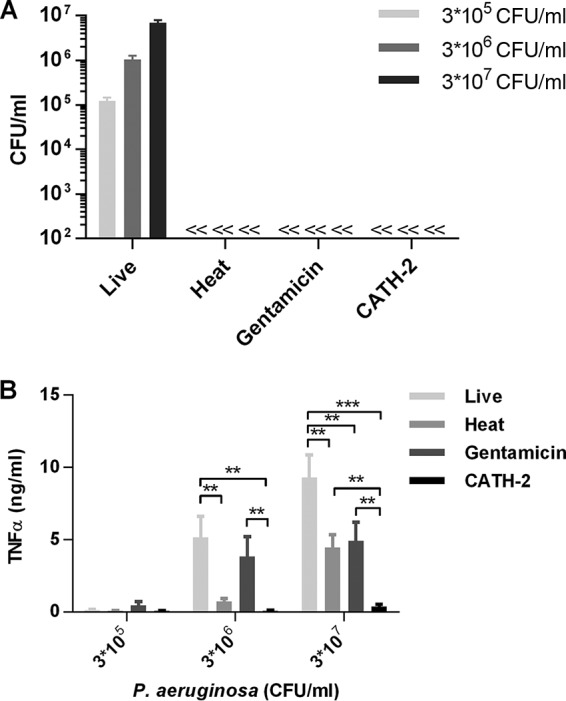
CATH-2 silently kills P. aeruginosa. Various concentrations of P. aeruginosa cells were left untreated or were CATH-2 killed, heat killed, or gentamicin killed and used for colony counting assays (A) or stimulation of J774.A1 cells for 2 h (B), after which TNF-α release was determined. Statistical differences were determined by two-way ANOVA with Bonferroni's *post hoc* test. *, *P* < 0.05; **, *P* < 0.01; ***, *P* < 0.001. *n* = 3. Error bars = SEM.

**FIG 3:**
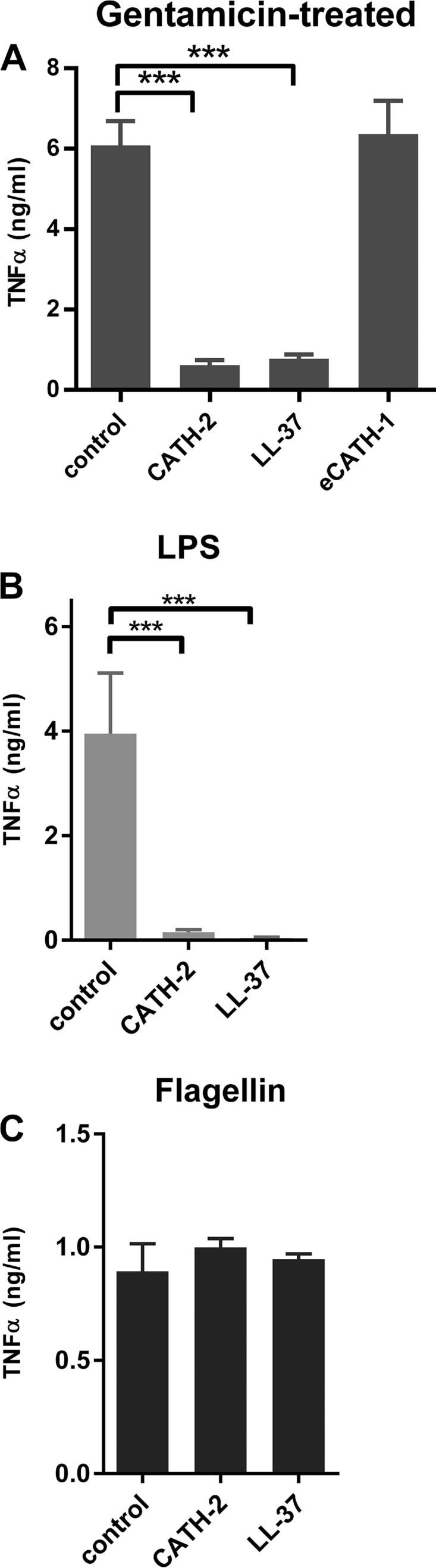
CATH-2 inhibits LPS-induced macrophage activation. (A) P. aeruginosa (3 × 10^6^ cells/ml) was incubated for 0.5 h with 250 μg/ml gentamicin, followed by the addition of 5 μM CATH-2, LL-37, or eCATH-1. These mixtures were used for stimulation of J774.A1 cells for 2 h, after which TNF-α release was determined. Statistical differences were determined by one-way ANOVA with Dunnett's *post hoc* test. *, *P* < 0.05; **, *P* < 0.01; ***, *P* < 0.001. *n* = 4. Error bars = SEM. (B and C) J774.A1 cells were stimulated with P. aeruginosa LPS (100 ng/ml) (B) or P. aeruginosa flagellin (10 ng/ml) (C) in combination with CATH-2 or LL-37, after which TNF-α release was determined after 2 h. *n* = 3. Error bars = SEM.

To determine the effect of bacterial killing on macrophage activation, P. aeruginosa either was left untreated or was heat killed, gentamicin killed, or CATH-2 killed (Fig. 2A). Subsequently, J774.A1 macrophages were stimulated for 2 h, after which TNF-α release was determined (Fig. 2B). Live and gentamicin-killed bacteria induced similar levels of TNF-α release at 3 × 10^6^ CFU/ml, while live P. aeruginosa is more potent at 3 × 10^7^ CFU/ml than gentamicin-killed bacteria. Heat-killed P. aeruginosa did not induce TNF-α release below 3 × 10^7^ CFU/ml, while CATH-2-mediated killing almost completely inhibited TNF-α release at all bacterial concentrations, indicating that CATH-2-mediated killing of P. aeruginosa is immunologically silent. Because both CATH-2 and LL-37 were previously shown to inhibit the activation of macrophages by nonviable E. coli (19), macrophages were also stimulated with gentamicin-treated bacteria (250 μg/ml) in combination with CATH-2, LL-37, or eCATH-1, after which TNF-α production was measured (Fig. 3A). Similar to the previously described results with E. coli, both CATH-2 and LL-37 were able to inhibit macrophage activation by gentamicin-treated P. aeruginosa, while eCATH-1 did not affect activation. Furthermore, to determine whether the inhibition is related to the inhibition of TLR activation, J774.A1 macrophages were stimulated with the known P. aeruginosa-derived TLR ligands LPS (Fig. 3B) and flagellin (Fig. 3C) in the presence of 5 μM CATH-2 or LL-37. While LPS-induced TNF-α production was potently inhibited by both CATH-2 and LL-37, flagellin-induced activation was unaffected by either peptide.

### CATH-2 inhibits P. aeruginosa-induced PMN recruitment *in vivo*.

**FIG 4:**
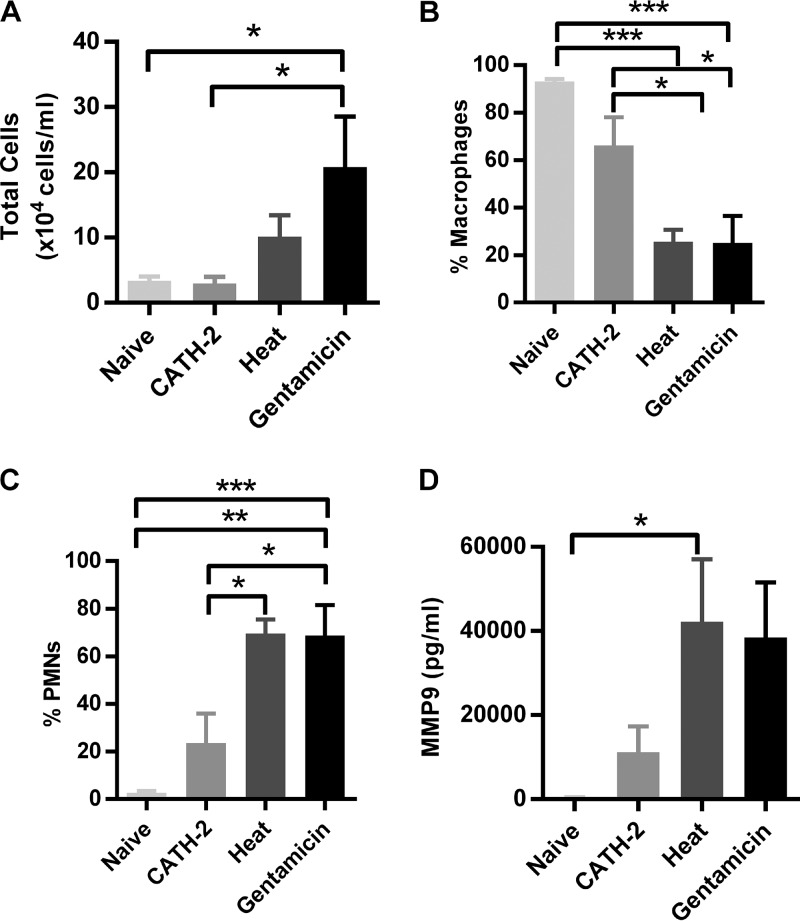
CATH-2-mediated killing prevents *in vivo* lung inflammation. Male C57BL/6 mice were intratracheally instilled with 50 μl of 2 × 10^6^ CFU/ml P. aeruginosa, which was either CATH-2 killed (20 μM), gentamicin killed (1 mg/ml), or heat killed (90°C, 1 h). Alternatively, control mice were instilled with an air bolus. After 6 h, total cell counts in BALF (A), as well as macrophage (B) and PMN (C) percentages, were determined by differential cell count. In addition, MMP-9 concentrations in BALF (D) were determined. *n* = 5 or more. Error bars = SEM. Statistical differences were determined by one-way ANOVA with Bonferroni's *post hoc* test. *, *P* < 0.05; **, *P* < 0.01; ***, *P* < 0.001.

The results described above show that CATH-2 is able to inhibit *in vitro* macrophage activation against P. aeruginosa; however, it is unknown whether this inhibitory effect is maintained in an *in vivo* setting. To determine whether this is the case, heat-killed, gentamicin-killed, or CATH-2-killed P. aeruginosa (2 × 10^6^ CFU/ml) was instilled in mouse lungs for 6 h, after which lung function was assessed and leukocyte numbers, cytokine/chemokine release, and total protein content were determined in BALF. Lung compliance and elastance were determined to assess whether the different experimental conditions would affect lung function; however, no significant changes were observed (Table 1). Analysis of total cell numbers in the BALF showed that the killing of P. aeruginosa by gentamicin resulted in the highest cell count (Fig. 4A). Treatment with heat-killed bacteria also increased cell numbers in the BALF, albeit not significantly, and no change in total cell numbers was observed in animals after treatment with CATH-2-killed P. aeruginosa, in comparison to naive animals. In both the heat-killed and gentamicin-killed treatment groups, polymorphonuclear cells (PMNs) were the main cell type in BALF, while macrophages remained the largest portion of cells in the naive mice and mice treated with CATH-2-killed bacteria (Fig. 4B and C), although mice treated with CATH-2-killed bacteria did show a nonsignificant increase in PMNs compared to the naive mice (PMN counts: naive, 0.09 ± 0.04 cells/ml; CATH-2 treated, 0.68 ± 0.75 cells/ml; heat treated, 7.41 ± 3.10 cells/ml; gentamicin treated, 18.85 ± 6.63 ×10^4^ cells/ml). In addition, the number of PMNs in the treatment groups correlated with the higher matrix metallopeptidase 9 (MMP-9) levels measured in the BALF (Fig. 4D), which has previously been linked to PMN influx (20). Furthermore, no changes in the BALF protein content were detected after treatment with CATH-2-killed, heat-killed, or gentamicin-killed bacteria, although there was a tendency toward higher protein levels in the group that received CATH-2-killed bacteria (Table 1).

**TABLE 1:** Effect of CATH-2 treatment on lung function^*a*^

Treatment	Lung compliance (ml/cm H_2_O × 10^3^)	Lung elastance (cm H_2_O/ml)	Protein content (mg/kg BW)
Control	57.8 ± 7.0	17.5 ± 2.2	14.05 ± 1.8
CATH-2	52.9 ± 9.2	19.4 ± 3.6	21.6 ± 9.1
Heat	55.6 ± 8.0	18.3 ± 2.9	14.45 ± 3.1
Gentamicin	58.9 ± 8.7	17.3 ± 2.6	15.75 ± 6.2

a Male C57BL/6 mice were instilled with 50 μl of 2 × 10^6^ CFU/ml P. aeruginosa, which was either CATH-2 killed (20 μM), gentamicin killed (1 mg/ml), or heat killed (90°C, 1 h). Alternatively, control mice were instilled with an air bolus. After 6 h, lung compliance was determined and lung elastance was calculated. In addition, total protein levels in the BALF were determined. *n* = 3 or more. Statistical analysis was performed by one-way ANOVA with Bonferroni's *post hoc* test, but no significant differences were detected.

### CATH-2 inhibits P. aeruginosa-induced cytokine and chemokine secretion *in vivo*.

**FIG 5:**
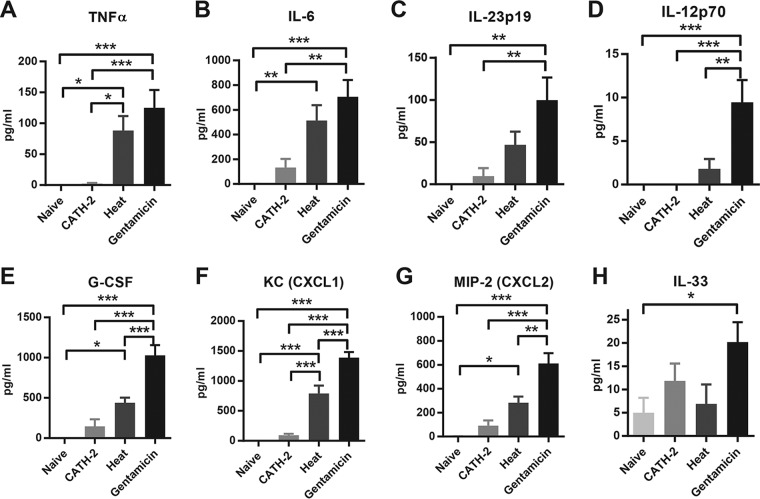
Effect of CATH-2-mediated killing on proinflammatory cytokine release *in vivo*. Male C57BL/6 mice were instilled with 50 μl of 2 × 10^6^ CFU/ml P. aeruginosa, which was either CATH-2 killed (20 μM), gentamicin killed (1 mg/ml), or heat killed (90°C, 1 h). Alternatively, control mice were instilled with an air bolus. After 6 h, TNF-α (A), IL-6 (B), IL23p19 (C), IL-12p70 (D), G-CSF (E), KC (F), MIP-2 (G), and IL-33 (H) were determined by Luminex technology. *n* = 5 or more. Error bars = SEM. Statistical differences were determined by one-way ANOVA with Bonferroni's *post hoc* test. *, *P* < 0.05; **, *P* < 0.01; ***, *P* < 0.001.

To further examine the extent of inflammation in the lung, multiplex analysis was performed on various pro- and anti-inflammatory cytokines, as well as various chemokines. Both heat-killed and gentamicin-killed bacteria induced the release of proinflammatory cytokines TNF-α (Fig. 5A) and IL-6 (Fig. 5B), while gentamicin-killed bacteria also significantly induced the release of IL-23p19 (Fig. 5C) and IL-12p70 (Fig. 5D) into the BALF. Treatment with CATH-2-killed bacteria resulted in significantly lower concentrations of TNF-α, IL-6, IL-23p19, and IL-12p70 than did treatment with gentamicin-killed bacteria and did not induce a significant increase in these cytokines compared to those in naive mice (Fig. 5A to D). Similar induction patterns were observed for granulocyte colony-stimulating factor (G-CSF) (Fig. 5E), keratinocyte chemoattractant (KC) (Fig. 5F), and macrophage inflammatory protein 2 (MIP-2) (Fig. 5G), with gentamicin-killed P. aeruginosa being the strongest inducer of cytokine release, followed by heat-killed P. aeruginosa. Treatment with CATH-2-killed P. aeruginosa resulted in values close to those obtained for naive mice, and these values were significantly lower than the cytokine release induced by treatment with gentamicin-killed P. aeruginosa. Furthermore, IL-33 was significantly increased only in the gentamicin-killed treatment group (Fig. 5H). Levels of IL-1β, IL-4, IL-10, and monocyte chemoattractant protein 1 (MCP-1) remained low and did not show any significant changes (data not shown).

### CATH-2 inhibits inflammation induced by P. aeruginosa killed by gentamicin *in vivo*.

**FIG 6:**
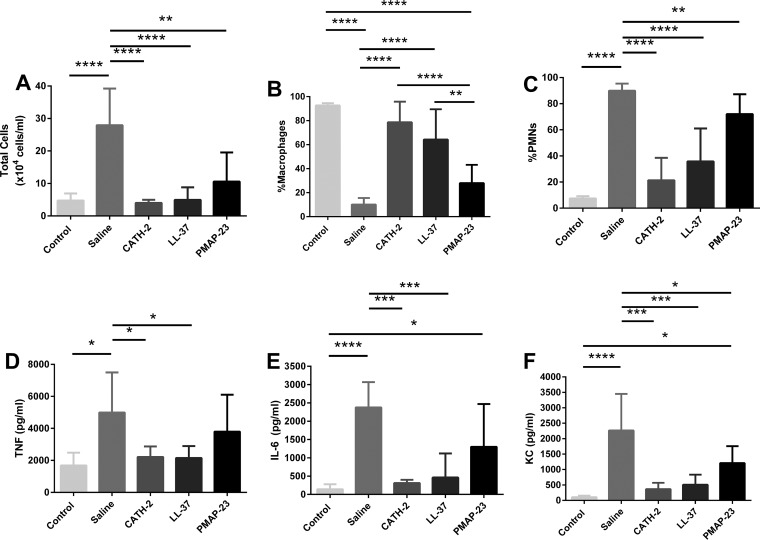
Effect of cathelicidins on inflammation induced by gentamicin-killed bacteria *in vivo*. Male C57BL/6 mice were instilled with 50 μl of 2 × 10^6^ CFU/ml P. aeruginosa killed by gentamicin (4 μg/ml), which was supplemented with either CATH-2, LL-37, or PMAP-23 at 20 μM, or were instilled without cathelicidin supplementation (saline). Control mice received 50 μl of saline containing gentamicin only. After 6 h, total cell counts in BALF (A), as well as macrophage (B) and PMN (C) percentages, were determined by differential cell count, and TNF-α (D), IL-6 (E), and KC (F) concentrations in the lavage fluid were determined by ELISA. *n* = 5 or more. Error bars = SEM. Statistical differences were determined by one-way ANOVA with Bonferroni's *post hoc* test. *, *P* < 0.05; **, *P* < 0.01; ***, *P* < 0.001.

To further explore the anti-inflammatory effects of CATH-2 that are independent of its killing activity, CATH-2, LL-37, or porcine myeloid antimicrobial peptide-23 (PMAP-23) was added to gentamicin-killed bacteria and instilled into the mouse lung. Six hours following the instillation, lung lavages were performed and the BALF was analyzed for differential cell counts and for three of the inflammatory mediators, TNF-α, IL-6, and KC, that showed a marked response in the previous experiment (Fig. 5). Compared to control (gentamicin-instilled) animals, animals receiving gentamicin-killed P. aeruginosa had a significant inflammatory response, as indicated by a large increase in the total cell count, percentage of neutrophils, and the concentrations of the three inflammatory cytokines in the lavage fluid (Fig. 6A to F). Treatment with gentamicin-killed P. aeruginosa that was supplemented with 20 μM CATH-2 or LL-37 resulted in a significantly reduced total cell count and percentage of neutrophils, and this was associated with significantly lower concentrations of TNF-α, IL-6, and KC (Fig. 6A to F). Administration of gentamicin-killed P. aeruginosa that was supplemented with the non-LPS binding peptide PMAP-23 resulted in an intermediate response for each of the outcomes (Fig. 6A to F).

## DISCUSSION

CATH-2 has previously been shown to cause “silent killing,” i.e., to kill bacteria in a nonimmunogenic manner (19). The current study provides evidence that silent killing by CATH-2 *in vitro* is not restricted to E. coli and also occurs against P. aeruginosa, a clinically relevant lung pathogen. In addition, our results provide evidence that CATH-2-mediated killing of P. aeruginosa inhibits pulmonary inflammation in a mouse lung model by reducing PMN recruitment and preventing the release of proinflammatory cytokines and chemokines. Based on these data, it is concluded that CATH-2 kills Gram-negative bacteria in an immunogenically silent manner, limiting inflammation both *in vitro* and *in vivo*.

While CATH-2 potently inhibits P. aeruginosa-induced macrophage activation, it also strongly inhibits TLR4 activation by P. aeruginosa LPS. This is in line with a previous study, which shows that silent killing of E. coli by CATH-2 is a two-step process in which CATH-2 first kills E. coli and then subsequently neutralizes LPS from the bacterial outer membrane to inhibit TLR4 activation (19, 21). In contrast, killing of P. aeruginosa by gentamicin, a clinically relevant antibiotic for pneumonia, or, alternatively, by heat treatment was not sufficient to prevent an inflammatory response. For E. coli, it was shown that these methods cause very different morphological changes in E. coli with subsequent differing levels of release of bacterial products (mainly LPS for Gram-negative bacteria), which can lead to an immune response (19), as observed for P. aeruginosa in the current study. Our results also show that while LL-37 is unable to kill P. aeruginosa under cell culture conditions, it can inhibit macrophage activation by gentamicin-treated P. aeruginosa and inhibit TLR4 activation by P. aeruginosa LPS, since the non-LPS binding peptide eCATH-1 did not possess this activity (Fig. 3A and B). This corresponds to the previously reported lack of antimicrobial activity of LL-37 against E. coli under cell culture conditions and the inhibition of macrophage activation by LL-37 in the context of nonviable E. coli only (19, 22). Together, this strongly suggests that both CATH-2 and LL-37 inhibit P. aeruginosa- and E. coli-induced macrophage activation through similar mechanisms but that only CATH-2 has the dual function of killing Gram-negatives under physiological cell culture conditions and subsequently inhibiting macrophage activation.

Notably, these *in vitro* observations were recapitulated in the *in vivo* studies. Whereas the *in vitro* environment provides strong evidence for the role of CATH-2 in limiting macrophage inflammation in the presence of bacterial products, an *in vivo* environment is more complex, as inflammation will involve multiple cell types, cell migration, and a physiological local milieu that may vary between different airways and alveoli. Despite this complexity, *in vivo* administration of CATH-2-killed P. aeruginosa resulted in significantly less inflammation, including reduced neutrophil infiltration and lower concentrations of various inflammatory cytokines, compared to that of heat- or gentamicin-killed bacteria. Further, LL-37 was also able to downregulate inflammation *in vivo* when added to gentamicin-killed bacteria. Based on the *in vitro* observations, the mechanism for these effects of both CATH-2 and LL-37 is through limiting TLR4 activation; however, other mechanisms cannot be excluded, specifically since PMAP-23, which lacks LPS binding capability, still reduced inflammation, although to a lesser extent than the other two peptides. Overall, these observations provide evidence for an important role of cathelicidins in mitigating inflammation due to bacterial products that are released when bacteria are killed *in vivo*.

Clinically, the anti-inflammatory effect of CATH-2 on TLR4 activation may have a strong potential benefit for the development of cathelicidin-based anti-infective therapies for CF patients. In these patients, TLR4-mediated immune activation has been shown to play an important role in inflammation during P. aeruginosa infections. This is partially caused by adaptations of P. aeruginosa to the environment of CF patients' lungs. It has been shown that P. aeruginosa modifies its LPS from a penta- to a hexa-acylated form, which is more potent in the activation of TLR4 (4, 23–25). In addition, regulation of immune activation, including TLR4 activation in alveolar macrophages and epithelial cells, appears to be dysregulated in CF patients, in part due to the lack of a functional cystic fibrosis transmembrane conductance regulator (CFTR) (26–28). This dysregulation includes the lack of proper TLR4 degradation in lysosomal compartments (29), as well as a lack of negative feedback upon TLR4 activation (30–32), which ultimately causes a higher inflammatory response in the lungs of CF patients. Since CATH-2 has a dual function of both killing P. aeruginosa and inhibiting TLR4 activation, treatment of P. aeruginosa infections in CF patients with CATH-2 (or CATH-2-derived compounds) can potentially reduce bacterial numbers and limit inflammation in the lung.

Another important characteristic of CATH-2 for anti-infective drug development is its antimicrobial activity under complex conditions, which includes solutions containing salt and serum components or bovine lipid extract surfactant (18, 33). Furthermore, CATH-2 has broad-spectrum antimicrobial activity, which includes activity against MDR P. aeruginosa (15) as well as activity against Staphylococcus aureus, which is another common infectious pathogen in CF patients (34). While no proof of silent killing of Gram-positives, such as S. aureus, is yet available, CATH-2 has been shown to inhibit macrophage TLR2 activation by S. aureus-derived lipoteichoic acid (19), suggesting that silent killing might not be restricted to Gram-negatives.

While our study has focused on the ability of CATH-2 to both kill bacteria and mitigate inflammation, other cathelicidins may also have potential beneficial functions in the treatment of lung infections. Our experiments suggest that LL-37 may have benefits as an anti-inflammatory agent that can be combined with antibiotics. In addition, while LL-37 is unable to directly kill P. aeruginosa under physiological conditions, a recent report showed that LL-37 can lower P. aeruginosa bacterial loads in a murine lung model, which appeared to be the result of increased PMN influx in the lung (35). This indicates that indirect effects can also play an important role in cathelicidin-mediated bacterial clearance from the lung and that different cathelicidins might depend on different functionalities to improve the outcome of infections. Furthermore, CATH-2-derived peptides, as well as other cathelicidins, have been shown to exert anti-biofilm activity, which could be important in the context of biofilm formation in CF patients (36–38). However, further research is needed to determine if these beneficial properties of cathelicidins can be translated into effective therapies for pneumonia and other infections. Specifically related to our study, future studies should examine the silent killing and potential other effects of CATH-2 in the context of an *in vivo* P. aeruginosa infection under CF-like conditions.

As with all studies, our experiments are associated with some limitations. For example, our *in vivo* studies were limited to studying anti-inflammatory effects due to bacterial products of killed bacteria. The advantage of this approach was to solely assess the anti-inflammatory effects of the peptides independent of their bactericidal activities; the ability of CATH-2, and potentially other peptides, to both kill bacteria and mitigate inflammation *in vivo* requires further study. In addition, a minor limitation, due to logistical issues, was that the *in vitro* experiments utilized eCATH1, but the *in vivo* study used PMAP-23, as a non-LPS binding peptide. However, it should be noted that both of these peptides have been extensively characterized with regard to their activities (18).

Overall, our results provide evidence for the silent killing of a relevant lung pathogen by CATH-2. While silent killing by CATH-2 has been observed against E. coli and Salmonella Enteritidis *in vitro*, this is the first study that shows that CATH-2-mediated killing of P. aeruginosa leads to inhibition of inflammation *in vitro* as well as *in vivo*. Together with previous reports, these results underline the potential for CATH-2 as a template for the development of an anti-infective therapy, for instance for CF patients, with both antimicrobial and anti-inflammatory functions.

## MATERIALS AND METHODS

### Reagents.

P. aeruginosa LPS was obtained from Sigma-Aldrich (St. Louis, MO, USA), and P. aeruginosa flagellin was obtained from Invivogen (Toulouse, France). Chicken CATH-2, human LL-37, and porcine myeloid antimicrobial peptide-23, (PMAP-23) were synthesized by Fmoc (9-fluorenylmethoxy carbonyl) chemistry at China Peptides (CPC Scientific, Sunnyvale, CA, USA), and equine CATH-1 was synthesized by Fmoc chemistry at the Academic Centre for Dentistry Amsterdam (Amsterdam, the Netherlands). Gentamicin solution was obtained from Sigma-Aldrich.

### Bacterial culture.

For *in vitro* experiments, P. aeruginosa ATCC 27853 (ATCC, Manassas, VA, USA) was grown to log phase in Luria broth (BioTRADING Benelux B.V., Mijdrecht, the Netherlands). After measurement of the optical density (OD), bacteria were centrifuged at 1,200 × *g* for 10 min and diluted in DMEM (Thermo Fisher Scientific, Waltham, MA, USA). To prepare killed bacteria, bacteria were incubated for 1 h at 90°C (heat killed), 1 h with 1 mg/ml gentamicin at 37°C (gentamicin killed), or 1 h with 20 μM CATH-2 at 37°C (CATH-2 killed).

### Cell culture.

J774.A1 murine macrophages were a kind gift of Jos van Putten (Division of Infection Biology, Department of Infectious Diseases and Immunology, Utrecht University, the Netherlands). Cells were cultured in DMEM supplemented with 10% FCS (Bodinco B.V., Alkmaar, the Netherlands). Cells were seeded in 96-well plates (7.5 × 10^4^ cells/well) for adherence overnight. Cells were subsequently stimulated with live, heat-killed, gentamicin-killed, or CATH-2-killed bacteria in the presence or absence of other cathelicidins. After 2 h of stimulation, TNF-α concentrations were determined in the supernatant. Alternatively, to determine IL-6 concentrations in the supernatant, cells were washed three times after 2 h of incubation, followed by an additional 22 h of incubation in the presence of 250 μg/ml gentamicin.

### ELISA.

ELISA DuoSets for mouse TNF-α and mouse IL-6 were obtained from R&D Systems (Minneapolis, MN, USA). Samples were diluted in phosphate-buffered saline (PBS) with 1% bovine serum albumin (BSA), pH 7.4, before analysis. ELISAs were performed according to the manufacturer's protocol. For ELISA plate analysis, absorbance was determined at an OD at 450 nm (OD_450_) and was corrected at OD_570_. Absorbance was determined with a FLUOstar Omega microplate reader (BMG Labtech GmbH, Ortenberg, Germany) and analyzed with MARS data analysis software (BMG Labtech GmbH).

### Colony counting assay.

Colony counting assays were performed after coincubation of P. aeruginosa with cathelicidins in 20-μl volumes at 37°C for 2 h in round-bottom polypropylene 96-well plates. After incubation, samples were diluted with 180 μl PBS, which was followed by spread-plating 10-fold dilutions in PBS on tryptic soy agar (TSA) plates (Oxoid Limited, Hampshire, United Kingdom). Plates were incubated overnight at 37°C, after which CFU counts were determined, with a detection limit of 10^2^ CFU/ml.

### Preparation of killed bacteria for *in vivo* analysis.

An overnight culture of P. aeruginosa ATCC 27853 was diluted 10-fold in tryptic soy broth (TSB). The optical density was measured, and bacteria were further diluted in sterile saline to approximately 2 × 10^6^ CFU/ml. Subsequently, the bacteria were killed by CATH-2, heat, or gentamicin as described above and immediately used without any additional wash steps for intratracheal instillation. Part of the bacterial solution was plated via spot plating on TSA and incubated overnight at 37°C to ensure complete bacterial killing. For our second *in vivo* experiment, a similar procedure was utilized except that the bacteria were killed by incubation with a lower dose of gentamicin (4 μg/ml).

### Administration of killed bacteria *in vivo*.

Male C57BL/6 mice (Charles River, Sherbrooke, Quebec, Canada), weighing 23 to 32 g, were used for this experiment. All animal procedures were approved by the Animal Use Subcommittee at the University of Western Ontario and followed the approved guidelines described by the Canadian Council of Animal Care. Mice were anesthetized by intraperitoneal (i.p.) injection of ketamine (130 mg/kg body weight [BW]) and dexmedetomidine (0.5 mg/kg BW) and then intubated using a 20-gauge catheter, with the aid of a fiber optic stylet (BioLite intubation system for small rodents; BioTex, Inc., Houston, TX, USA). Once intubated, the mice were randomized to instillation with 50 μl of heat-, gentamicin-, or CATH-2-killed bacterial preparations (see above) or were instilled with an air bolus (naive controls). Animals were randomized to a specific administration, and experiments were performed on 3 or 4 mice per day (different treatment groups on the same day). Animals were housed individually after intratracheal instillation, and individual mice were used for the statistics. Mice were extubated following successful instillation and subsequently injected with a reversal agent for dexmedetomidine, atipamezole (Antisedan), and allowed to breathe spontaneously for the following 6 h. After 6 h, the mice were euthanized by i.p. injection of sodium pentobarbital and dissection of the descending aorta. The animals were placed on a FlexiVent system to measure lung compliance and elastance. Following these measurements, whole-lung lavage fluid was collected by using three 1-ml aliquots of sterile saline. The whole-lung lavage fluid was immediately centrifuged at 150 × *g* at 4°C for 10 min, and the pellet was collected for cell analysis, while the supernatant was collected to measure protein content and cytokine concentrations. Differential cell analysis of the cells obtained in the lavage fluid was done as previously described (39). Protein content of the lavage fluid was measured using a Micro BCA protein assay kit (Pierce, Rockford, IL, USA), according to the manufacturer's instructions. Levels of mouse cytokines were measured using multiplexed immunoassay kits according to the manufacturer's instructions (R&D Systems, Minneapolis, MN). A Bio-Plex 200 readout system (Bio-Rad) was used, which utilizes Luminex xMAP fluorescent bead-based technology (Luminex Corporation, Austin, TX). Cytokine levels were automatically calculated from standard curves using Bio-Plex Manager software (v.4.1.1; Bio-Rad).

In a second experiment, similar procedures were utilized; however, intubated mice were randomized to receive 50 μl of gentamicin-killed bacterial preparations without or with supplementation with 20 μM CATH-2, LL-37, or PMAP-23. A group of mice receiving 50 μl of gentamicin (4 μg/ml in saline) was used as a control group. Six hours following intubation, the mice were euthanized and lung lavage was performed as described above. Differential cell analysis of the cells obtained in the lavage fluid was done as previously described above. The levels of mouse IL-6, KC, and TNF-α were measured using ELISA kits per the manufacturer's instructions (R&D Systems, Minneapolis, MN). A Bio-Plex 200 readout system was used (Bio-Rad), and cytokine levels were automatically calculated from standard curves using Bio-Plex Manager software (v.4.1.1, Bio-Rad).
